# Inhibitory Effects of *Eurotium cristatum* on Growth and Aflatoxin B_1_ Biosynthesis in *Aspergillus flavus*

**DOI:** 10.3389/fmicb.2020.00921

**Published:** 2020-05-15

**Authors:** Qiannan Zhao, Yue Qiu, Xin Wang, Yuanyuan Gu, Yuzhu Zhao, Yidi Wang, Tianli Yue, Yahong Yuan

**Affiliations:** ^1^College of Food Science and Engineering, Northwest A&F University, Yangling, China; ^2^Laboratory of Quality & Safety Risk Assessment for Agro-products, Ministry of Agriculture, Yangling, China; ^3^College of Food Science and Technology, Northwest University, Xi’an, China; ^4^College of Enology, Northwest A&F University, Yangling, China

**Keywords:** *Eurotium cristatum*, aflatoxin B_1_, *Aspergillus flavus*, gene expression, degradation products

## Abstract

Probiotic strain *Eurotium cristatum* was isolated from Chinese Fuzhuan brick-tea and tested for its *in vitro* activity against aflatoxigenic *Aspergillus flavus*. Results indicated that *E. cristatum* can inhibit the radial growth of *A. flavus.* Furthermore, this inhibition might be caused by *E. cristatum* secondary metabolites. The ability of culture filtrate of strain *E. cristatum* against growth and aflatoxin B_1_ production by toxigenic *A. flavus* was evaluated *in vitro*. Meanwhile, the influence of filtrate on spore morphology of *A. flavus* was analyzed by scanning electron microscopy (SEM). Results demonstrated that both radial growth of *A. flavus* and aflatoxin B_1_ production were significantly weakened following increases in the *E. cristatum* culture filtrate concentration. In addition, SEM showed that the culture filtrate seriously damaged hyphae morphology. Gas chromatography mass spectrometry (GC/MS) analysis of the *E. cristatum* culture supernatant revealed the presence of multiple antifungal compounds. Real-time quantitative polymerase chain reaction (RT-qPCR) analysis showed that the expression of aflatoxin biosynthesis-related genes (*aflD*, *aflQ*, and *aflS*) were down-regulated. Importantly, this latter occurrence resulted in a reduction of the AflS/AflR ratio. Interestingly, cell-free supernatants of *E. cristatum* facilitated the effective degradation of aflatoxin B_1_. In addition, two degradation products of aflatoxin B_1_ lacking the toxic and carcinogenic lactone ring were identified. A toxicity study on the HepG2 cells showed that the degradation compounds were less toxic when compared with AFB_1_.

## Introduction

Aflatoxins, toxic derivatives of difuran coumarin, are predominantly synthesized by *Aspergillus* species such as *Aspergillus flavus* and *Aspergillus parasiticus* (Karabulut et al., 2014). Several forms of aflatoxin exist with aflatoxin B_1_ (AFB_1_) being the most toxic (Adebo et al., 2017; Gong et al., 2019). Aflatoxin B_1_ poses serious problems to human health and is known to cause acute or chronic toxicity aflatoxicosis resulting in mutagenic, carcinogenic, teratogenic, and immunosuppressive effects in humans (Sherif et al., 2009; Bbosa et al., 2013; Santini and Ritieni, 2013). It is estimated that aflatoxins may play a causative role in 4.6–28.2% of all global hepatocellular carcinoma cases (Liu and Wu, 2010). AFB_1_ has been classified as Group I carcinogen to humans by the International Agency for Research on Cancer (IARC, 1993). In addition to causing serious health issues, aflatoxins pollution in food and feed results in significant economic losses (Wu, 2015). Thus, multifarious physical (such as artificial removal, physisorption, temperature, and humidity control) and chemical (such as alkali and oxidation treatments, chemical agents) methods have been developed to alleviate associated problems in recent decades (Torres et al., 2014; Ismail et al., 2018). However, these methods not only consume large amounts of energy, but also significantly reduce the nutritional efficiency of food while adversely impacting available natural resources (Wang et al., 2015; Peng et al., 2018). Moreover, with the excessive application of synthetic fungicides as well as the potential for extended exposure, numerous fungi have developed resistance (Pham et al., 2014; Hawkins et al., 2019). For the afore-mentioned reasons, an effective alternative is urgently required.

Bio-control agents represent an attractive alternative in relation to the reduction or elimination of aflatoxin contamination (Tsitsigiannis et al., 2012; Medina-Cordova et al., 2016; Nguyen et al., 2017). These strategies are beneficial as they offer milder means of removing or degrading toxic materials; they can also ensure that significant losses in aesthetic and nutritional value are ameliorated. Previous studies have reported that some bacterial or fungal strains including *Bacillus subtilis*, *Nocardia asteroides*, *Rhodococcus erythropolis*, and *Aspergillus niger* can inhibit aflatoxins (Chaudhary et al., 2001; Wu et al., 2009; Cserhati et al., 2013; Zhang et al., 2017). And the most promising method for reducing contamination levels of aflatoxins is introduction of non-aflatoxin producing strains to soil where they compete with wild-type aflatoxigenic populations (Jaime-Garcia and Cotty, 2007; Wu and Khlangwiset, 2010; Alshannaq et al., 2018). A non-toxigenic *A. flavus* Afla-Guard (NRRL 21882) isolated by Dorner and Lamb (2006) have been used commercially on the peanuts in the southeastern United States. In addition, the atoxigenic *A. flavus* K49 (NRRL 30797, isolated from Maize) and AF36 (NRRL 18543, isolated from Cottonseed) also have been registered as a biopesticide for the management of aflatoxin-producing fungi during the crop production (Abbas et al., 2011). Although these strains could reduce aflatoxin contamination effectively, they still face significant challenge (Ehrlich, 2014). One of the major problems is a potential risk of introducing a heavy dose of *A. flavus* strains. The diversity of *A. flavus* populations may trigger other toxic secondary metabolites even imposed an additional burden on food safety and food quality especially with global warming (Alshannaq et al., 2018). Thus, it is imperative that identify other effective strains for the treatment of aflatoxin contamination.

*Eurotium cristatum*, which has been commonly known as “golden flower,” is used as a non-toxic and safe fungus for food fermentation (such as dark tea, okara, kudzu root) (Yazdani et al., 2011; Zhang et al., 2018; Chan et al., 2019; Gu et al., 2019). And it is the main probiotic fungus traditionally used in Fuzhuan brick dark tea in China (Peng et al., 2011; Shi et al., 2019). Actually, some studies suggesting that *E. cristatum* was useful in regulating the blood/lipid balance and cholesterol metabolism, enhancing immunity, alleviating obesity, and modulating gut microbiota (Liu et al., 2016; Chen et al., 2018; Du et al., 2019; Kang et al., 2019). Moreover, previous studies have reported that filtrate of *E. cristatum* exhibits significant inhibitory activity on some bacterial or fungal strains (such as *Staphylococcus aureus*, *Escherichia coli*, and *Magnaporthe grisea*) (Xu et al., 2015; Du et al., 2017). Therefore, in this study, a probiotic strain of *E. cristatum* isolated from Fuzhuan tea was used for the first time to control *A. flavus*.

Here, we evaluated the ability of *E. cristatum* isolated from Fuzhuan tea to inhibit the *in vitro* production of AFB_1_ by toxigenic *A. flavus*. The results of this study will provide scientists with a better understanding of the mechanisms underpinning aflatoxin inhibition by *E. cristatum*.

## Materials and Methods

### Chemicals and Fungal Strains

Aflatoxin B_1_ standard was purchased from Shanghai Yuanye Bio-Technology Co., Ltd., China. And other chemicals were purchased from Sigma-Aldrich, United States.

A single *E. cristatum* strain HNYYWX.21 was isolated from Fuzhuan brick-tea in our laboratory (Wang et al., 2019). The AFB_1_-producing *A. flavus* strain used in this study was originally isolated from natural spices and kept in our laboratory. These strains were grown on potato dextrose agar (PDA) medium (potato 200 g/L, glucose 20 g/L, agar 15 g/L) for 7 days at 28 ± 2°C until good sporulation was observed. Conidial suspensions (1 × 10^7^ conidia/mL) were prepared from sporulated cultures with sterile 0.01% (vol/vol) Tween 80.

### Growth Curves of *A. flavus* and *E. cristatum*

Ten microliters of spore suspension (1 × 10^6^ spores/mL) of *A. flavus* and 10 μL with 1 × 10^7^ spores/mL of *E. cristatum* were inoculated on PDA medium for 5 days at 28 ± 2°C, respectively. The growth curve was obtained by measuring the colony diameter every 24 h. All treatments were repeated three times.

### Preparation of the *E. cristatum* Culture Filtrate

The *E. cristatum* culture filtrate was prepared according to a method described by Xing et al. (2017) with minor modifications. Fifty milliliters of potato dextrose broth (PDB) was inoculated [2% (v/v) inoculum] with a conidial suspension (10^7^ spores/mL) at 28 ± 2°C in a rotary shaker incubator (120 r/min) for 10 days. The culture filtrate was obtained following centrifugation at 10,000 *g* for 10 min at 4°C and subsequent passage through a 0.45-μm pore size filter (Millipore, United States); the filtrate was stored at −20°C until further required.

### *In vitro* Antifungal Assay

#### Competition Assay on PDA Plates

An antagonism experiment was performed using relative growth of *A. flavus*. Briefly, 10 μL of an *A. flavus* fungal suspension at a concentration of 10^6^ spores/mL were inoculated onto the center of a PDA plate, and 10 μL of the *E. cristatum* (10^7^ spores/mL) suspension and sterile water (used as control) were independently inoculated on 5-mm diameter sterile filter paper discs that were positioned 25 mm from the center of the plate. The plates were incubated at 28 ± 2°C and the radial growth of each fungal colony was measured by a digital caliper every 24 h until the control group was overgrown with agar plate. All experiments were repeated for three times.

The inhibition ratio (%) = (*r*−*r*′)/*r* × 100%, where *r* (mm) represents the radius of the *A. flavus* colony (from the center to the control group) and *r*′ (mm) represents the growth of the *A. flavus* colony from the center toward the *E. cristatum* colony.

#### Effect of the *E. cristatum* Culture Filtrate on *A. flavus* Growth and AFB_1_ Production

The influence of the *E. cristatum* culture filtrate on the radial growth of the *A. flavus* mycelium was assayed according to a method described by Xu et al. (2013) with some minor modifications. The *E. cristatum* culture filtrate was adjusted to concentrations of 10, 20. and 40% (v/v) with PDA, respectively. Ten microliters of the *A. flavus* conidial suspension (at a concentration of 10^6^ spores/mL) was inoculated onto each plate; the plates were incubated in the darkness at 28 ± 2°C for 5 days. Equal volume of water replaced culture filtrate served as a control. The diameter of the *A. flavus* colony was assayed every 24 h and the level of AFB_1_ in the PDA medium was analyzed after the culture period. All treatments were repeated three times.

#### Effect of the Culture Filtrate on the Ultrastructure of *A. flavus*

Potato dextrose broth (10 mL) containing culture filtrate [40% (v/v)] was inoculated with *A. flavus* conidia [10^6^ spores/mL, 2% (vol/vol)] and incubated at 28 ± 2°C in a rotary shaker (120 r/min). PDB with water[(40% (v/v)] was used as a control. All treatments were repeated three times. The germination of *A. flavus* spores was investigated by scanning electron microscopy (SEM) after 24 h according to a method published by Sangmanee and Hongpattarakere (2014). After incubation, fungal mycelia were washed four times (10 min per wash) with sterile water. Next, the fungal mycelia were fixed overnight at 4°C with 2.5% glutaraldehyde. The samples were subsequently washed with sterile water to remove free glutaraldehyde; this was followed by dehydration in a graded series of ethanol (10, 30, 50, 70, 85, 95, 100, and 100%) for 10 min. The samples were then dried in a critical point drier (CPD) with liquid carbon dioxide, coated with gold in a polaron sputter coater and examined using a Nova Nano SEM-450 (FEI, United States).

### Effect of the Culture Filtrate on the Expression of Genes Associated With AFB_1_ Production

Ten microliters of the *A. flavus* conidial suspension (at a concentration of 10^6^ spores/mL) was inoculated into 50 mL of PDB containing 30% (v/v) of the culture filtrate. PDB with 30% (v/v) of water was used as a control group. They were incubated in the dark at 28 ± 2°C on a rotary shaker at 120 r/min in triplicate. After 3 days, the expression of genes associated with AFB_1_ production were assayed by real-time quantitative polymerase chain reaction (RT-qPCR).

#### Total RNA Extraction

RNA was isolated from fungal hyphae after 3 days. Approximately 100 mg of ground mycelium in liquid N_2_ was treated with 1 mL of Trizol (Sigma-Aldrich) according to the manufacturer’s instructions. RNA samples were treated with RNase-Free DNase (QIAGEN, Germany) to remove genomic DNA. The concentration and purity (A260/A280 ratio) of RNA were determined by a NanoDrop spectrophotometer (IMPLEN, Germany). RNA samples with A260/A280 values ranging from 1.97 (control group) to 2.05 (*E. cristatum* culture filtrate group) were used in this study. RNA was detected by 1% agarose gel electrophoresis, until the appearances of 5S band, clear 28S and clear 18S. Then the purified RNA was stored at −80°C until further analysis.

#### cDNA Synthesis

Reverse transcription was performed using the Fastking RT Kit (with gDNase) (QIAGEN, Germany). First-strand cDNA was obtained from 1 μg of total RNA in a 20-μL reaction mixture. Each reaction mixture was incubated at 42°C for 15 min followed by 95°C for 3 min and the product was stored at −20°C until further required.

#### Real-Time PCR

In this study, RT-qPCR was performed in a LightCycler96 detection system (Roche, Switzerland). Primers for genes associated with AFB_1_ such as *aflR*, *aflS*, *aflD*, *aflQ*, *VeA*, *LaeA*, and β*-tubulin* (as an internal control) used in this study were listed in Table 1, and they were used to understand the relationship between aflatoxin biosynthesis and the active compound of *E. cristatum* culture filtrate. Three replicates of both template free-negative control and endogenous control were used for each run. Each RT-qPCR reaction consisted of 10 μL of SuperReal PreMix Color (SYBR Green) (QIAGEN, Germany), different concentrations of each primer, and 100 ng of cDNA template in a final volume of 20 μL. Cycling conditions were as follows: 95°C for 15 min, 95°C for 10 s, 58°C (56°C for *aflQ*) for 30 s, 72°C for 32 s (40 cycles) in a 20-μL reaction mixture. A melt curve was generated at the end of every run to ensure PCR product uniformity by heating at 95°C for 10 s, 65°C for 60 s and 97°C for 1 s. All samples were analyzed in triplicate and qPCR reactions were repeated three times.

**TABLE 1:** Nucleotide sequences of primers for RT-qPCR assays.

Gene	Nucleotide sequence (5′-3′)	References	Concentration (μM)	Melting temperature (°C)	Product size (bp)	Accession number
β*-tubulin*	F: TCTTCATGGTTGGCTTCGCT	Peromingo et al., 2017	6	88.39	98	FR775333.1
	R: CTTGGGGTCGAACATCTGCT		6			
*aflR*	F: GATCTGGCTGGTCAGGAGCA	Chen et al., 2019	6	89.57	/	/
	R: CGCCTGAAACGGTGGTAGTG		6			
*aflS*	F: TGGTGCGACCATATTTACA	Peromingo et al., 2017	6	85.81	94	AF441435.2
	R: GGTTGGGTCACGAACTGTTT		6			
*aflD*	F: ATGCTCCCGTCCTACTGTTT	Liang et al., 2015	6	88.32	108	XM_002379908
	R: ATGTTGGTGATGGTGCTGAT		6			
*aflQ*	F: TTAAGGCAGCGGAATACAAG	Moon et al., 2016	8	90.32	/	/
	R: GACGCCCAAAGCCGAACACAAA		8			
*VeA*	F: TTGTCGTGTGCGGATTCG	Cary et al., 2007	6	86.51	/	/
	R: CTCATCGTAGTCGTAGTCATCG		6			
*LaeA*	F: AAAGGTTGCTCGCTGGTACA	Liu et al., 2017	6	84.65	121	/
	R: GACTTCTGACGAAATGCGCC		6			

*The symbol “/” indicates that it is not mentioned in the reference.*

#### Relative Gene Expression

Relative quantification of the expressions of the *aflR*, *aflS*, *aflD*, *aflQ*, *VeA*, and *LaeA* genes were done compared to the housekeeping gene β*-tubulin.* Data analysis was performed using the 2^–ΔΔ*Ct*^ analysis method (Livak and Schmittgen, 2001). The PCR efficiency of each genes were practically equal since the amplification curves of the target and reference genes were parallel to each other in the exponential amplification interval. The specificity of the reactions was checked by analyzing the melt curves, which displayed a single sharp peak (Luo et al., 2005).

### AFB_1_ Degradation by the *E. cristatum* Culture Filtrate

The effect of the culture filtrate of *E. cristatum* on AFB_1_ was studied according to a method published by Rao et al. (2017). One microliter of AFB_1_ standard was added to 1 mL of the culture filtrate until a final concentration of 1000 ng/mL was achieved; this mixture was subsequently incubated at 28 ± 2°C in the dark in a shaker incubator at 200 r/min for 0, 3, and 5 days. One milliliter of the PDB (with 1000 ng/mL AFB_1_) and the *E. cristatum* culture filtrate as the internal control were incubated under same conditions, respectively. All treatments were repeated three times. The remnant AFB_1_ and associated metabolites in the liquid medium were analyzed by high performance liquid chromatography (HPLC) and liquid chromatography quadrupole time of flight-mass spectrometry (LC-qTOF/MS).

### Analysis of AFB_1_ by HPLC

AFB_1_ determination was performed using a Shimadzu LC-20A equipped with a fluorescence detector (RF-20A). AFB_1_ accumulated in the culture media was extracted according to a method by Zhou et al. (2017) with some modifications. Briefly, 2 g of PDA medium (1 mL of liquid medium) was placed in a 4-mL Eppendorf tube and 1 mL of chloroform was added. The mixture was blended at 3200 r/min for 90 s and sonicated (100 watts) for 20 min; the sample was subsequently centrifuged for 10 min at 8000 r/min. This procedure was repeated three times and the organic layer containing AFB_1_ was combined and then evaporated to dryness under an N_2_ stream at 50°C. The derivatizing reaction was performed by adding 200 μL of n-hexane and 100 μL of trifluoroacetic acid (TFA) for 1 h at 45°C in the darkness. After evaporating to dryness once more and redissolution in a 1000-μL mixture of 2/8 (v/v) acetonitrile/water, the mixture was violently shaken for 2 min and sonicated for 20 min at 100 watts of power. Next, each solution was filtered through a sterile 0.22-μm pore size filter (Millipore, United States) before injection into the HPLC. A series of reference compounds (10, 20, 50, 100, and 1000 ng/mL) at different concentrations was used to quantify the AFB_1_ content.

High performance liquid chromatography analysis of AFB_1_ was performed as follows: a XDB-C_18_ column (4.6 × 250 mm, 5 μm, Agilent, United States) was operated at 40°C with a flow rate of 1 mL/min with acetonitrile-water as mobile phase. A starting mobile phase of 20% acetonitrile was orderly escalated to 30% within 13 min, then the gradient elution was switched to 40% over 5 min and this was subsequently maintained for 1 min. Next, the eluted ratio returned to 20% once more over 2 min, and this ratio was maintained for 7 min. The injection volume was 20 μL. The excitation and detection wavelengths were 360 and 440 nm, respectively. The calibration curves for AFB_1_ (10–1000 ng/mL) by HPLC revealed a good linear relationship (*R*^2^ ≥ 0.99) between the detector response and the amounts of the AFB_1_ standards. The limit of detection (LOD) obtained in this study were 0.27 ng/mL.

### Analysis of AFB_1_ Degradation Products by LC-qTOF/MS

Samples were extracted three times using equal volumes of chloroform. The samples were subsequently dried with N_2_ and re-dissolved in acetonitrile/water (2/8, v/v). LC/MS was performed on an LC-30A + SelexION + TripleTOF5600 + system (AB SCIEX, United States) equipped with a Agilent Plus C_18_ column (2.1 × 150 mm, 5 μm). The mobile phase for elution was composed of 70% acetonitrile (0.1% formic acid in water) with a flow rate of 0.4 mL/min. The total run time was 32 min and the injection volume was 25 μL. MS was performed using the following conditions (Iram et al., 2016): positive-ion mode, the capillary voltage and temperature were 3.5 kV and 300°C, respectively. Nitrogen was used as the collision gas. LC/MS analysis was performed to identify any potential degraded products in a full-scan mode within the range of m/z 100–2000.

### Evaluation of the *E. cristatum* Culture Filtrate by GC/MS

Prior to extraction, the culture filtrate of *E. cristatum* to which 40 ng/mL 4-methyl-1-pentanol (internal standard) was added was equilibrated at 40°C for 30 min. PDB as the control group was incubated under same conditions, and all experiments were carried out three times independently. Gas chromatography mass spectrometry (GC/MS) was performed on a GC/MS-QP2010 Ultra system (Shimadzu, Japan). Chromatographic separation was performed on a DB-1MS column (60 m × 0.25 mm ID, 0.25 μm film thickness). Helium was used as the carrier gas with a flow rate of 1 mL/min. An inlet temperature of 40°C with splitless injection was employed. The temperature program was as follows: 40°C for 3 min; 4°C/min ramp to 120°C; 6°C/min ramp to 240°C and subsequent hold for 2 min. The total run time was 55 min. MS was performed using the following conditions: ion source temperature 230°C, transfer line temperature 230°C, scan mass range 35–500 amu, solvent delay time 3 min. The assay was subsequently performed according to the internal standard method.

### Cytotoxicity Studies

#### HepG2 Cell Culture and Preparation of Samples

The human hepatocyte carcinoma HepG2 cell line was cultured in Dulbecco’s modified Eagle’s medium (10% fetal bovine serum, 1% non-essential amino acids, 100 U/mL penicillin, 100 μg/mL streptomycin) (D5796, Sigma-Aldrich) at 37°C in a 5% CO_2_ humidified atmosphere. The AFB_1_ degradation products (20 mL) and AFB_1_ stock solution (20 mL of 1 μg/mL of AFB_1_) were dried with N_2_ and re-dissolved in 0.1% (v/v) dimethyl sulfoxide (DMSO). Untreated AFB_1_ stock solution was used as the control group. And HepG2 cells grew under normal conditions and treated with DMSO [0.1% (v/v)] as the nulling group (internal control) and blank group, respectively. All experiments were repeated six times.

#### MTT Assay

MTT assay, a way to detect cell proliferation, was performed as described by Adebo et al. (2016) with some modifications. Briefly, 100 μL of HepG2 culture at a density of 1 × 10^3^ cells/mL was pipetted into 96-well plates. After overnight stabilization, cells were treated in 100 μL of fresh culture medium with appropriate concentrations of the tested compounds and then incubated for 48 h. Next, 10 μL of MTT [50 mg/mL; 3-(4,5-dimethylthiazol-2-yl)-2,5-diphenyltetrazolium bromide] were added to the culture medium and the plates were incubated for a further 4 h. All the culture conditions were at 37°C in a 5% CO_2_ humidified atmosphere. After removing the culture medium, 150 μL of DMSO was added to the plates. The plates were subsequently incubated for 10 min in a rotary shaker incubator (120 r/min) in the darkness and the absorbance was read in a victor X3 (Perkin Elmer, United States) at a wavelength of 490 nm.

Cell viability = (OD_e_−OD_b_)/(OD_n_−OD_b_) × 100%, where OD_e_ is the absorbance of the experimental group (cells with AFB_1_ stock solution and AFB_1_ degradation products); OD_n_ is the absorbance of the nulling group; OD_b_ is the absorbance of the blank group.

### Statistical Analysis

Data analysis was performed by Student’s *t*-test and ANOVA using IBM SPSS V20 (SPSS/IBM, Chicago, IL, United States). The means for each treatment were separated by Tukey’s test using a level of significance of 0.05.

## Results and Discussion

### Antagonistic Assay of *E. cristatum* Against *A. flavus*

**FIGURE 1:**
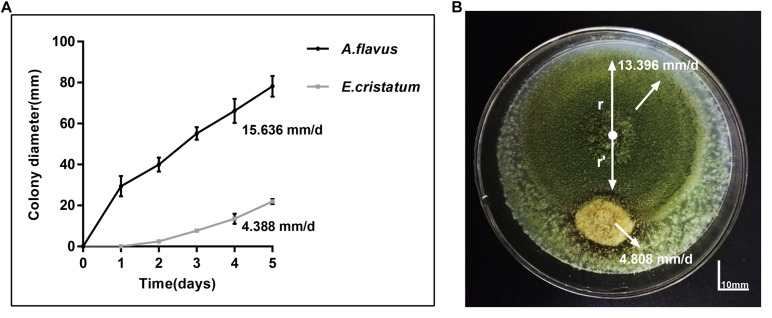
Confronting incubation assay and growth curve for *A. flavus* and *E. cristatum.* **(A)** Growth curves of *A. flavus* and *E. cristatum* in PDA at 28 ± 2°C for 5 days. Data spots represent mean values of three determinations ± standard deviation. **(B)** *A. flavus* was inoculated at the center of the PDA plate and *E. cristatum* and sterile water (control) were inoculated at 25 mm from the center of the plate.

*Aspergillus flavus* and *E. cristatum* were co-cultured on PDA plates for 5 days. *E. cristatum* exhibited mycelial growth inhibition against *A. flavus* (Figure 1B) with an inhibition ratio of 63.74%. The inhibition of fungal growth might be attributed to competition for nutrients and/or space or the synthesis of inhibitory metabolites. However, the average growth rate of *E. cristatum* (4.39 mm/day) was obviously lower than that of *A. flavus* (15.64 mm/day) under the same conditions (*p* < 0.05, Figure 1A). Shi et al. (2019) have reported that metabolites produced by *E. cristatum* inhibit the growth of *B. subtilis*. Similarly, Du et al. (2014) observed that metabolites of *E. cristatum* such as cristatumin A, cristatumin D, and 3-O-(a-D-ribofuranosyl) might be active against some bacterias and fungi (such as *B. subtilis*, *S. aureus*, and *M. grisea*). Thus, we speculate that the inhibition of *E. cristatum* on *A. flavus* also might be caused by the synthesis of secondary metabolites by *E. cristatum*. Hence, further studies were required to determine the effects of *E. cristatum* culture supernatant.

### Effect of the *E. cristatum* Culture Filtrate on Growth and Aflatoxin Accumulation in *A. flavus*

**FIGURE 2:**
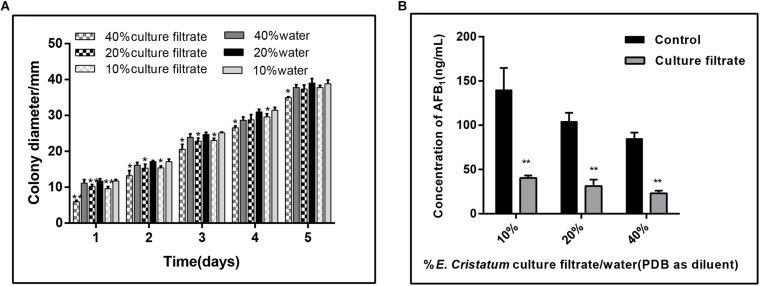
Effects of the *E. cristatum* culture filtrate on growth and AFB_1_ production of *A. flavus*. **(A)** The colony diameter of *A. flavus* in PDA medium with 10, 20, and 40% *E. cristatum* culture filtrate or water at 28 ± 2°C for the first 5 days. **(B)** AFB_1_ content in PDA medium with different concentrations of *E. cristatum* culture filtrate and water at 28 ± 2°C for 5 days. Values in each column followed by one (*p* < 0.05) or two (*p* < 0.01) asterisks are significantly different from the control according to the Student’s *t*-test.

To test whether *E. cristatum* secretes unknown compound(s) into medium that confer *A. flavus* inhibition, we filter-sterilized (0.45 μm filter) the 10-day-old culture of *E. cristatum* grown in PDB, and combined the cell-free culture with *A. flavus* and 40 mL of fresh PDA. As a control, a set of water were mixed with the PDA. *A. flavus* grown on PDA plates with different concentrations of the *E. cristatum* culture filtrate and water were observed every 24 h. As shown in Figure 2A, the radial growth of *A. flavus* was significantly inhibited by the *E. cristatum* culture filtrate (*p* < 0.05). And the ratios of inhibition were ranged from 5.90 to 44.17%. Xu et al. (2013) reported that a 2% (v/v) of 10-fold concentrated the culture filtrate of strain *A. niger* (FS10) inhibits the growth of *A. flavus* (49.6%) while impeding AFB_1_ production (94.5%). The latter study also revealed that there is a direct correlation between biological growth and AFB_1_ production. Thus, in this study, the effect of the *E. cristatum* culture filtrate on aflatoxin production in PDA medium was also determined on the seventh day. According to the results shown in Figure 2B, the AFB_1_ production was significantly (*p* < 0.01) reduced from 139.04 to 40.41 ng/mL when the PDA medium contained only 10% culture filtrate. The AFB_1_ enormously decreased from 84.87 to 23.07 ng/mL, and resulting in a 72.52% inhibition when the culture filtrate concentration was 40%. similarly, Alshannaq et al. (2018) reported that the culture filtrate of strain *Aspergillus oryzae* M2040 in concentrations of 10 and 25% (V:V in water or PDB) could significantly inhibit *A. flavus* NRRL 3357 spore recovery and AFB_1_ production (*p* < 0.01). Although Shi et al. (2019) and Zhang et al. (2019) have demonstrated that *E. cristatum* exhibits antimicrobial activity, this study is the first to report on the inhibition of aflatoxigenic *A. flavus*.

### Fungal Morphology Following Scanning Electron Microscopy

**FIGURE 3:**
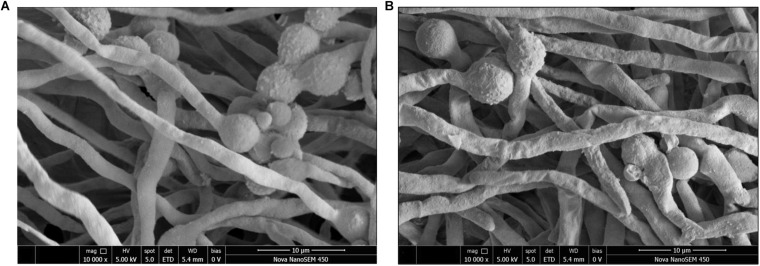
Morphology of *A. flavus* spores inoculated in PDB medium with the *E. cristatum* culture filtrate **(B)** and water **(A)**.

The effects of the *E. cristatum* culture filtrate on spore germination and morphology of *A. flavus* were studied by SEM after 24 h. Typical SEM images of treated and untreated (PDB with water) spore suspensions are depicted in Figure 3. Importantly, the *E. cristatum* culture filtrate did not affect spore germination, while fungal morphology was affected. This result is in accordance with a study published by Sangmanee and Hongpattarakere (2014). Complete mycelial structures often exhibit increased resistance to antimicrobial treatment (Liang et al., 2019). Sun et al. (2016) concluded that the antifungal effect of some the associated compounds may be partly ascribed to damage caused to the hyphal cell structure. As illustrated in Figure 3A, untreated *A. flavus* hyphae retained an elongated structure with a smooth appearance. Conversely, as shown in Figure 3B, the hyphae of the culture filtrate treatment were wrinkled and folded. Similar phenomena have been observed when *A. flavus* were treated with volatile organic compounds from *Streptomyces alboflavus* TD-1 (Yang et al., 2019) and *Streptomyces yanglinensis* 3–10 (Shakeel et al., 2018). This shriveled morphology might be attributed to interactions between components of the culture filtrate of *E. cristatum* and *A. flavus* and the cell walls; it is possible that associated interactions lead to visible membrane invagination.

### Gene Expression Studies

**FIGURE 4:**
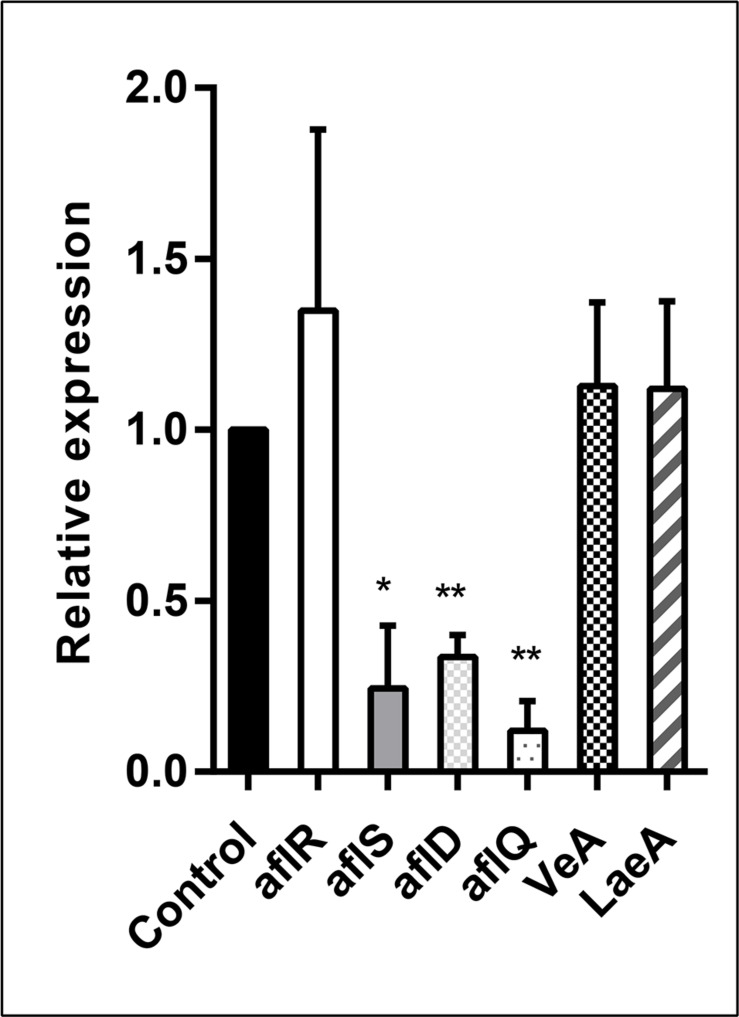
Relative expression values of the *aflR*, *aflS*, *aflD*, *aflQ*, *VeA*, and *LaeA* genes in *A. flavus* at 28 ± 2°C after 3 days. The β*-tubulin* gene was used as an internal control to normalize the expression data. The error bars represent the standard deviations from three independent experiments with three replicates each. Values in each column followed by one (*p* < 0.05) or two (*p* < 0.01) asterisks are significantly different from the control according to the Student’s *t*-test.

In order to analyze the effect of the *E. cristatum* culture filtrate on aflatoxin gene activation, *A. flavus* was grown in PDB containing 30% (v/v) *E. cristatum* culture filtrate for 3 days. Six genes (*aflR, aflS, aflD, aflQ, VeA*, and *LaeA*) were investigated relatively to reference gene (β*-tubulin*). As shown in Figure 4, the *E. cristatum* culture filtrate repressed the expressions of *aflD* (2.94-fold), *aflQ* (8.33-fold), and *aflS* (4.16-fold). *LaeA*, *veA*, and *aflR* gene expressions were not significantly impacted by the culture filtrate. The *aflR* gene is known to encode a major transcriptional regulator of aflatoxin biosynthesis genes, while *aflS* might be involved in the regulation of aflatoxin biosynthesis through the regulation of other genes (Tominaga et al., 2006; Wang B. et al., 2017). The *aflD* gene encodes the first stable intermediate norsolorinic acid during aflatoxin formation (Abdel-Hadi et al., 2010), while *aflQ* encodes an oxidoreductase, which is required for the final steps associated with the conversion of sterigmatocystin to AFB_1_ (Cary et al., 2012). In this study, the transcript level of *aflR* did not obviously change, while that of *aflS* was significantly downregulated (*p* < 0.05), and the ratio *aflR*:*aflS* was above 1. Upon normal expression of *aflR* and *aflS*, sufficient quantities of AflS protein combine with AflR protein to form the AflS (4)-AflR (1) complex; the formation of this complex results in the synthesis of natural levels of toxin (Kong et al., 2014). Here, the aflR/aflS balance was upset was following down-regulation of *aflS.* Although *aflR*:*aflS* ratio above one would lead to an activation of AFB_1_ biosynthesis (Schmidt-Heydt et al., 2009). In our study, a ratio above one was not correlated with high AFB_1_ accumulation. Similar results were also obtained by Verheecke et al. (2015). Additionally, expression of the two structural genes, *aflD* and *aflQ*, was also significantly reduced (*p* < 0.01). Quantitative PCR showed that lack of *aflS* transcript led to a reduction of *aflD* expression (Meyers et al., 1998). In addition, two- to four-folds reductions in a*flD*, *aflQ*, and *aflS* were observed when *A. flavus* had a reduction in AFB_1_ (Moon et al., 2018). Thus, the repressions of *aflS*, *aflD*, and *aflQ* expressions could be the main reasons for a reduction in the formation of AFB_1_ in our experimental conditions.

### Degradation of AFB_1_ in the *E. cristatum* Culture Filtrate at Different Exposure Times

**FIGURE 5:**
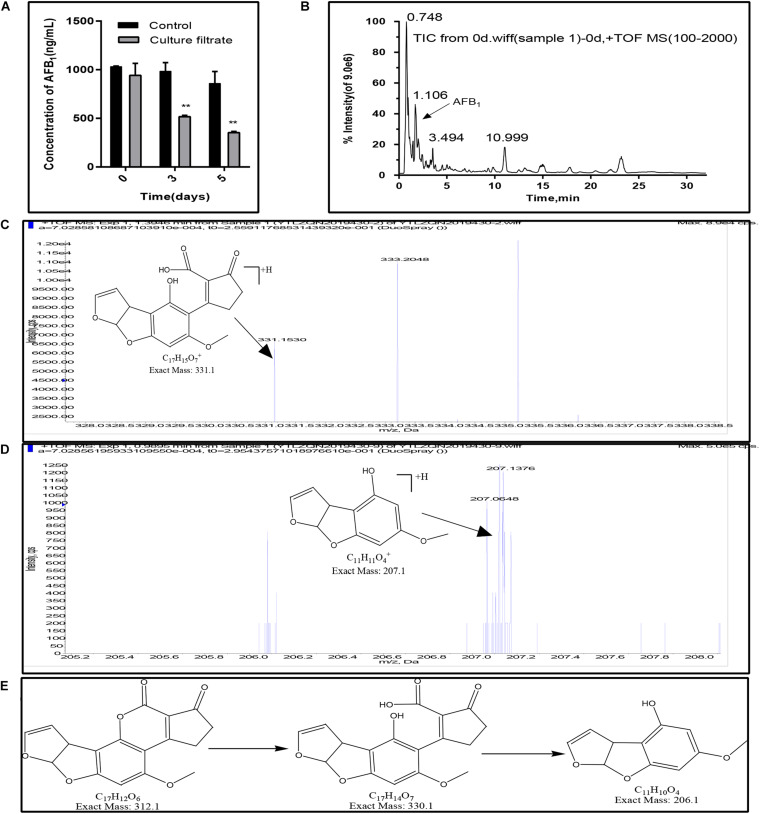
AFB_1_ degradation and fragmentation of biodegradation products of AFB_1_. **(A)** Residual aflatoxin B_1_ (ng/mL) in the *E. cristatum* culture filtrate and PDB over different time-periods at 28 ± 2°C. Error bars represent standard deviations of the means following three replicates each. **(B)** Electrospray ionization (ESI) total ion chromatogram (TIC) scan of control group (1000 ng/mL AFB_1_ in PDB broth, after incubation in the dark at 28 ± 2°C). **(C)** Putative degraded **Product 1**. **(D)** Putative degraded **Product 2**. **(E)** The deduced biodegradation pathway of AFB_1_.

In this study, the AFB_1_ degradation ability of the *E. cristatum* culture filtrate was confirmed by HPLC. As shown in Figure 5A, the concentration of AFB_1_ in the control were stable during the incubation time. And the total ion chromatograms of aflatoxin B1 of control group was showed in Figure 5B. However, the concentration of the AFB_1_ was reduced from 942.24 to 517.47 ng/mL when disposed the *E. cristatum* culture filtrate after 3 days. And the percentage of AFB_1_ degradation was approximately 47.42%. The microbial culture supernatants as the degrading matrix have been widely studied for their AFB_1_ degradation potential (Verheecke et al., 2016). The culture supernatants of *A. niger* D15-Lcc2#3, *Pleurotus ostreatus* St2-3, *Phanerochaete chrysosporium* ME-446, and *Bjerkandera adusta* SCC0169 also had the ability to degrade AFB_1_. AFB_1_ degradation rates by these culture filtrates were respectively, 55.0, 35.90, 13.77, and 28.19% after 3 days at 30°C (Alberts et al., 2009). In comparison, our 47.42% degradation rate was relatively high. Moreover, following increased incubation times, our result showed that the AFB_1_ degradation rate was 58.85%, and only 353.45 ng/mL of AFB_1_ was detected after 5 days (*p* < 0.01). This latter result indicates that the residual quantity of AFB_1_ was positively correlated with exposure times; this is in accordance with studies performed by Sangare et al. (2014) and Petchkongkaew et al. (2008). However, the supernatant of *Stenotrophomonas maltophilia* 35-3 was able to reduce 84.80% of AFB_1_ (initial concentration 2 mg/L) at 37°C when exposed a more longer time (90 h) (Guan et al., 2008). Therefore, further optimizations of degradation conditions (such as temperature, pH, metal ions) are necessary.

Furthermore, AFB_1_ degradation products have been observed and identified using LC-qTOF/MS. Compared with the control (AFB_1_ with PDB and just only the *E. cristatum* culture filtrate), two degraded products with suggested molecular formulae of **C_17_H_14_O_7_ (product 1)** and **C_11_H_10_O_4_ (product 2)** with [M + H]^+^ ion peaks at m/z 331.15 (Figure 5C) and 207.06 (Figure 5D) were detected in the treated samples following Aanalyst TF1.7.1 analysis. Generally, aflatoxicol, aflatoxin B_2__*a*_ (AFB_2__*a*_), and aflatoxin D_1_ (AFD_1_) were the most reported AFB_1_ degradation products. Nakazato et al. (1990) found that *Eurotium herbariorum* could convert AFB_1_ to aflatoxicol-A (AFL-A) by reducing the cyclopentenone carbonyl of AFB_1_, then AFL-A was converted to aflatoxicol-B (AFL-B) by the actions of medium components or organic acids produced from the fungi, this study also found that *A. niger* could convert AFB_1_ to AFB_2__*a*_ by producing organic acids to lowering pH of the medium. Eshelli et al. (2015) studied the metabolomics of the AFB_1_ degradation by the *R. erythropolis* culture filtrate. The author stated that AFB_1_ degradation may involve the formation of the β-keto acid structure, followed by hydrolysis of the lactone ring resulting in a metabolite with 330 amu (product 1 in this study). The hydrolysis was followed by decarboxylation of the open lactone ring yielding to AFD_1_, this process was also involved the formation of AFD_2_. In this study, as shown in Figure 5E, AFB_1_ degradation may involve the hydrolysis of the lactone ring resulting in **product 1** with 330 m/z, followed by decarboxylation of the open lactone ring yielding a product known as AFD_2_ with 206 m/z **(product 2)**, where the lactone carbonyl and cyclopentenone ring disappeared. Although the products still retained the 8,9-dihydrofuran double bond, the associated toxicities were much less than that of AFB_1_ (Eshelli et al., 2015).

### Composition of the *E. cristatum* Culture Filtrate

Putative metabolites (Table 2) of the *E. cristatum* culture filtrate were analyzed using GC/MS (PDB medium was used as the control). Numerous substances, which have been shown to elicit significant antimicrobial activities in previous studies, were observed in the present study. Of the volatile components detected, alcohols, including 1-hexanol and 1-octen-3-ol, were the predominant substances detected. Due to the fact that these materials non-selectively adsorbed and predominantly accumulated in the cell membrane, thereby inhibiting membrane function, it was deduced that these substances exhibit antimicrobial activity (Ingram and Buttke, 1984). 1-octen-3-ol at 4.87 ng/L was the most abundant of these substances observed in this study. (Zunino et al., 2015) found that 1-hexanol (4.24 mM) can effectively inhibit fumonisin production by *Fusarium verticillioides*. Haidar et al. (2016) reported that 1-octen-3-ol can strongly inhibit the growth of *Phaeomoniella chlamydospora*. Similarly, Xiong et al. (2017) revealed that 1-octen-3-ol can inhibit fungal growth and spore germination, while also suggesting that this compound can change the permeability of the cell membrane.

**TABLE 2:** Metabolites in the *E. cristatum* culture filtrate identified by GC/MS.

Number	RT	RI	Compounds	Concentrations (ng/L)
1	4.542	408	Acetaldehyde	0.10 ± 0.02
2	11.071	715	(*E*)-2-pentenal	0.66 ± 0.35
3	11.211	790	Acetylacetone	3.12 ± 2.96
4	15.19	860	1-hexanol	1.25 ± 0.30
5	20.233	969	1-octen-3-ol	4.87 ± 0.91
6	21.029	1005	Octanal	0.46 ± 0.00
7	22.926	1018	D-limonene	0.29 ± 0.02
8	23.448	1029	Acetophenone	0.30 ± 0.08
9	26.554	1059	1,2-dimethoxy-benzene	0.18 ± 0.06
10	27.093	1124	3,5,5-trimethyl-hexanoic acid	3.33 ± 1.94
11	31.849	1262	Thymol	0.93 ± 0.76
12	32.456	1293	(R)-(+)-Citronellic acid	0.39 ± 0.01
13	34.526	1331	Propanoic acid,2-methy, l,3-hydroxy-2,2,4-trimethylpentyl ester	1.15 ± 0.72
14	40.041	1605	Pentanoic-acid,2,2,4-trimethyl-3-carboxyisopropyl, isobutyl ester	1.32 ± 1.43

Aldehydes have stronger antifungal activity than alcohols (Ando et al., 2012). (*E*)-2-pentenal (0.66 ng/L), acetaldehyde (0.10 ng/L), and octanal (0.46 ng/L) were the predominant aldehydes analyzed. Previous studies have shown that acetaldehyde can inhibit the growth of *Rhizopus stolonifer*, *Botrytis cinerea, Alternaria alternata*, and *Penicillium digitatum* (Zhou et al., 2018). Wright et al. (2000) indicated that octanal (100 μL/L) had potent fungitoxicity against *A. parasiticus* with an inhibition ratio of 76%; this study also revealed that octanal also inhibited AFB_1_ production. In addition, thymol (0.93 ng/L), acetophenone (0.30 ng/L) and d-limonene (0.29 ng/L) also exhibited remarkable antimicrobial activities. A study by Lambert et al. (2001) suggested that thymol inhibits the anthracnose pathogen and is positively correlated with damage to the membrane; these occurrences affect pH homeostasis and the equilibrium of inorganic ions. Boukaew and Prasertsan (2018) previously reported on the inhibitory activities of acetophenone (100 μL/L) in relation to the growth and sporulation of both *A. flavus* TISTR 3041 and *A. parasiticus* TISTR 3276 on PDA plates. Our results reveal that the *E. cristatum* culture filtrate is bioactive and volatile-rich, and individual quantitative analysis of the inhibitory activities of these compounds will be required in future studies.

### Cell Viability Assay

**FIGURE 6:**
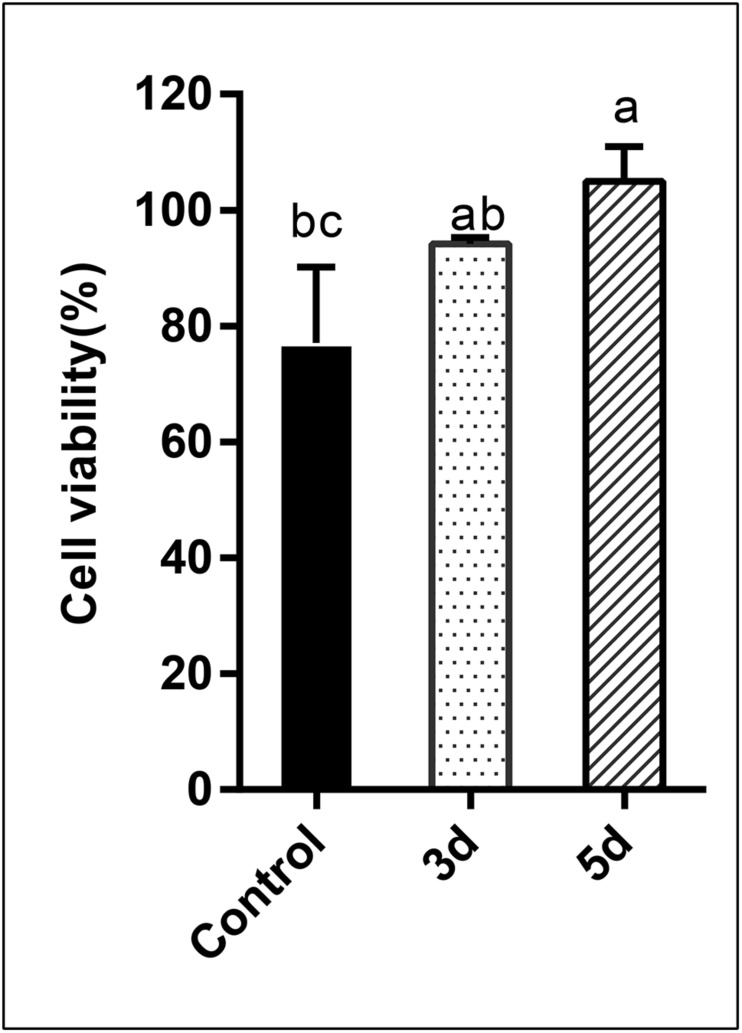
Analysis of the cytotoxicity of AFB_1_ degradation by cell-free *E. cristatum* culture filtrate using MTT assay. The error bars represent the standard deviations from three independent experiments with six replicates each. The same letters in each column indicate no significant differences among the data at the 5% level following the Tukey test.

In this study, the cytotoxicity of the AFB_1_ degraded products was assessed using human liver cancer cells via the MTT assay. Percentage cell viability is presented in Figure 6. Results indicated that the percentage cell viability was reduced to 77.10 and 94.17%, respectively, when cells were exposed to AFB_1_ stock solution and the AFB_1_ degradation (3 days) products. Conversely, when cells were treated with longer degradation periods (5 days) AFB_1_ extracts, evident growth-promoting effects (104.95%) were observed (*p* < 0.05). AFB_1_ is a well-documented hepatocarcinogen, and it can cause DNA damage by forming DNA-adducts, also can affect RNA translation and induce oxidative stress (Madalena et al., 2018). Since AFB_1_ is predominantly metabolized in the liver, the human hepatoma HepG2 cell model is considered to be the most suitable system for testing its *in vitro* toxicity (Domijan et al., 2019). Previous studies have showed that AFB_1_ could decrease HepG2 survival (Chan et al., 2003; Wang C. et al., 2017; Madalena et al., 2018). But in this study, AFB_1_ degradation extracts (5 days) stimulated the growth of HepG2 cells. Specifically, AFB_1_ has a two-phase effect on cells: low (0.5–1 μg/mL) doses promote the proliferation of HepG2 cells, while high doses inhibit proliferation (Wang C. et al., 2017). Therefore, our results suggest that AFB_1_ was degraded to relatively low levels by the *E. cristatum* culture filtrate. This occurrence may lead to the synthesis of other possibly less or non-toxic compounds. Furthermore, the cell-free culture extracts did not exhibit cytotoxicity to HepG2 cells and may even have stimulated the growth of HepG2 cells. Therefore, the degraded (5 days) AFB_1_ extracts exhibit increased stimulatory effects compared with AFB_1_ alone. Similar results were also observed by Ma et al. (2012).

## Conclusion

Our study showed that *E. cristatum* exhibited potential biocontrol activity against aflatoxigenic *A. flavus*. Thus, *E. cristatum* may represent an antagonist due to its capacity to reduce both fungal growth and AFB_1_ biosynthesis levels. GC/MS analysis indicated that there are many antifungal substances present in the *E. cristatum* culture filtrate. qRT-PCR analysis revealed a significant reduction in the AflS/AflR ratio. In addition, our results confirmed that atoxigenic *E. cristatum* could effectively degrade AFB_1_. Furthermore, two degradation products, where the lactone ring was destroyed, were identified by LC-qTOF/MS. In addition, cytotoxicity test showed that the degradation compounds were less toxic than AFB_1_. Further studies are required to investigate *E. cristatum* culture filtrate.

## Data Availability Statement

GenBank accession numbers for our nucleotide sequences: *A. flavus*: MN759628, HNYYWX.21: MN759629.

## Author Contributions

QZ, YQ, TY, and YY conceived and designed the experiments. QZ performed the experiments and analyzed the data. QZ and TY wrote the manuscript. XW, YG, YZ, and YW contributed reagents, materials, and analysis tools. All authors read and approved the final manuscript.

## Conflict of Interest

The authors declare that the research was conducted in the absence of any commercial or financial relationships that could be construed as a potential conflict of interest.
